# Strategy for Optimizing Vitamin B12 Production in *Pseudomonas putida* KT2440 Using Metabolic Modeling

**DOI:** 10.3390/metabo14110636

**Published:** 2024-11-18

**Authors:** Thomaz Satuye Prieto-de Lima, Keilor Rojas-Jimenez, Christopher Vaglio

**Affiliations:** 1 Postgraduate Program in Biology, University of Costa Rica, San José 11501, Costa Rica; 2 School of Biology, University of Costa Rica, San José 11501, Costa Rica; 3 Health Research Institute, University of Costa Rica, San José 11501, Costa Rica; christopher.vagliocedeno@ucr.ac.cr

**Keywords:** microbial biotechnology, metabolic engineering, genome-scale metabolic model, cobalamin, vitamin B12, flux balance analysis, *Pseudomonas putida* KT2440, systems biology

## Abstract

**Background/Objectives**: Vitamin B_12_ is very important for human health, as it is a cofactor for enzymatic activities and plays various roles in human physiology. It is highly valued in the pharmaceutical, food, and additive production industries. Some of the bacteria currently used for the vitamin production are difficult to modify with gene-editing tools and may have slow growth. We propose the use of the bacteria *Pseudomonas putida* KT2440 for the production of vitamin B_12_ because it has a robust chassis for genetic modifications. The present wok evaluates *P. putida* KT2440 as a host for vitamin B_12_ production and explore potential gene-editing optimization strategies. **Methods**: We curated and modified a genome-scale metabolic model of *Pseudomonas putida* KT2440 and evaluated different strategies to optimize vitamin B_12_ production using the knockin and OptGene algorithms from the COBRA Toolbox. Furthermore, we examined the presence of riboswitches as cis-regulatory elements and calculated theoretical biomass growth yields and vitamin B_12_ production using a flux balance analysis (FBA). **Results**: According to the flux balance analysis of *P. putida* KT2440 under culture conditions, the biomass production values could reach 1.802 gDW^−1^·h^1^·L^−1^, and vitamin B_12_ production could reach 0.359 µmol·gDW^−1^·h^−1^·L^−1^. The theoretical vitamin B_12_ synthesis rate calculated using *P. putida* KT2040 with two additional reactions was 14 times higher than that calculated using the control, *Pseudomonas denitrificans*, which has been used for the industrial production of this vitamin. **Conclusions**: We propose that, with the addition of aminopropanol linker genes and the modification of riboswitches, *P. putida* KT2440 may become a suitable host for the industrial production of vitamin B_12_.

## 1. Introduction

Among all vitamins, vitamin B_12_, also called cobalamin, is the largest and one of the most complex [1]; it is also an important nutrient and an essential cofactor for human enzymatic activities [2]. Vitamin B_12_ is used for DNA synthesis, erythrocyte production, and myelin maintenance. It also plays a vital role in mitochondrial metabolism and is important for preventing megaloblastic anemia [3]. Vitamin B_12_ production is currently of great interest to the pharmaceutical, food, and additive production industries [4]. In 2017, vitamin B_12_ was valued at approximately USD 2700 per kg of high purity [5]. Manufacturing this compound via chemical synthesis is extremely complex, as it involves a series of reactions of more than 60 steps, whereas, at the biological level, the vitamin is synthesized via the tetrapyrrole compound pathway, requiring about 30 enzyme-mediated steps [2,6].

Modified bacteria have significant advantages for producing natural compounds, as bacteria are easy to handle under laboratory conditions [7]. In addition, the efficient production of certain compounds can be optimized by introducing or modifying biosynthetic pathways with different genetic engineering strategies. In this regard, the use of metabolic models has improved the production yield of compounds of interest [8]. For these purposes, genome-scale metabolic models (GSMs) and constraint-based analyses have been developed, with flux balance analysis (FBA) being one of the most popular for metabolic simulations [9]. The FBA predicts theoretical metabolite production rate yields under different conditions, such as the available levels of oxygen, carbon sources, nitrogen sources, and amino acids [10,11]. Metabolic reconstructions at the genomic scale provide a platform to deepen the genomic, genetic, and biochemical knowledge of an organism, which can be transformed into a mathematical metabolic model [12]. Along with the FBA, methods such as OptGene are used to identify knockout gene targets that may enhance a biochemical process of interest, as well as biomass growth [13].

For the bacterium *Pseudomonas putida* KT2440, a metabolic model constructed with genomic, biochemical, and physiological information has been available since 2008 [14]. This Gram-negative soil bacterium has been “domesticated” for biotechnological purposes, and it has great potential in synthetic biology in particular [15]. The strain *P. putida* KT2440 has been recognized as a nonharmful strain and certified as “generally recognized as safe” (GRAS), facilitating laboratory studies as well as being suitable for industrial production [7]. Both *Pseudomonas putida* and *Pseudomonas denitrificans* have been recognized as microorganisms with vitamin B_12_ biosynthesis pathway genes [11].

In the case of *P. denitrificans*, the presence of B_12_-responsive riboswitches in mRNA is important for the vitamin B_12_ regulation process, as they work as mRNA control elements of metabolites sensing [16]. Riboswitches contain ligand-binding sensor domains that enable the alteration of gene expression at both the transcriptional and translational levels, as they can bind to metabolites such as vitamin derivatives [17]. A study by Nguyen-Vo et al. suggests that vitamin B_12_ biosynthesis can be considerably improved by modifying the promoter sequences regulated by riboswitches [16]. In this regard, the Rfam database can provide valuable information on noncoding RNA families that have these types of regulatory functions [18]. Currently, *P. denitrificans* is one of the species used for industrialized vitamin B_12_ production [2]. However, it has several limitations; for example, it has slow growth and is difficult to modify with gene-editing tools [4]. Conversely, *P. putida* KT2440 has optimal characteristics as a chassis [19].

This research aims to curate and modify a genome-scale metabolic model of *Pseudomonas putida* KT2440 to evaluate genetic engineering strategies for the optimization of vitamin B_12_ production. Using the algorithms present in the COBRA Toolbox, such as the gene knockin and OptGene algorithms, the metabolic model of *P. putida* KT2440 is modified, and different genetic modification strategies are evaluated to optimize vitamin B_12_ production. This analysis uses the biosynthetic production of *P. denitrificans* as a point of comparison. The presence of riboswitch sequences in the *P. putida* KT2440 genome, which may work as cis-regulatory elements of the metabolic pathway of vitamin B_12_, is also examined. The flux balance analysis is used to calculate theoretical biomass growth yields and vitamin B_12_ production yields in order to evaluate possible optimization strategies for vitamin B_12_ synthesis and thus propose a genetic circuit for biotechnological interventions.

### 1.1. Microbial Production of Vitamin B_12_

The overall process of vitamin B_12_ synthesis requires about 30 genes, and deleting any of them would result in the vitamin not being produced [20]. The cobalamin group has been found to be a part of the biosynthetic pathways in several microbial species. There are two major biological pathways for biosynthesis, namely, oxygen-dependent and oxygen-independent biological pathways [2], differing mainly in cobalt insertion time and oxygen requirements [4]. The aerobic route is present in organisms such as *Pseudomonas denitrificans*, *Sinorhizobium* (*Ensifer*) *meliloti*, *Rhodobacter sphaeroides*, and *Pseudomonas aeruginosa*. However, the anaerobic pathway is found in *Salmonella typhimurium*, *Klebsiella pneumoniae*, *Citrobacter amalonaticus*, *Bacillus megaterium*, *Propionibacterium shermanii,* and *Lactobacillus reuteri* [21].

**Figure 1:**
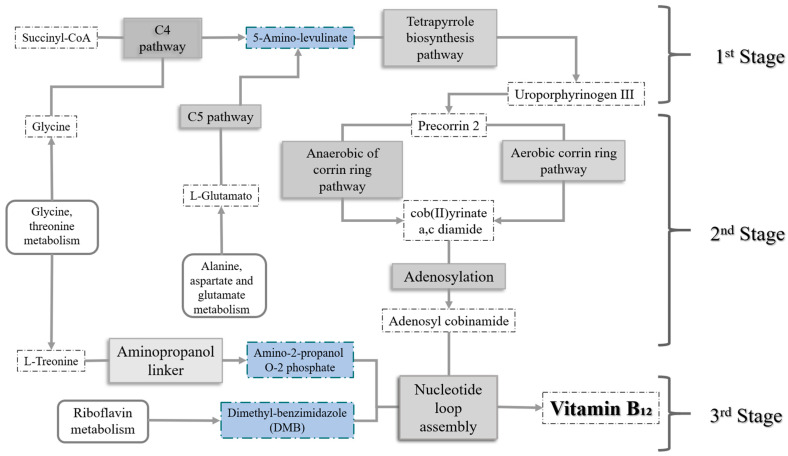
Main steps and stages in the metabolic synthesis pathway of vitamin B_12_. The major precursor compounds are highlighted. Adapted from [23].

Figure 1 shows the de novo production pathways, which comprise three major steps: (1) the production of uroporphyrinogen III (Uro III); (2) the transformation of UroIII into cobinamide (Cbi); and (3) the assembly of the nucleotide loop, which requires the synthesis of the lower axial ligand, usually 5,6-dimethylbenzimidazole [11]. Previously, the synthesis of 5-amino-levulinate (ALA) was also required, which can be produced by two pathways—the C4 and C5 pathways. In the C4 pathway, ALA is synthesized from glycine and succinyl-CoA with the enzyme ALA synthase (EC: 2.3.1.27). Conversely, the C5 pathway uses L-glutamate as a precursor, and three enzymatic reactions occur: Gltx (EC: 6.1.1.17), HemA (EC: 1.2.1.70), and HemL (EC: 5.4.3.8) [22]. *P. putida* KT2440 has the genes encoding the C5 pathway but lacks the ALA synthase corresponding to the C4 pathway [23].

In the first stage, from glycine to the synthesis of Uro III, 4 enzymes are involved; then, in the second stage, from Uro III to the synthesis of adenosyl cobinamide phosphate, 14 enzymes are involved, being the longest stage. Finally, in the last stage, vitamin B_12_ is produced from dimethylbenzimidazole (DMB) and the previously synthesized adenosyl cobinamide phosphate, for which four enzymes are involved: two are involved in the transformation of DMB; one is involved in the synthesis of adenosine GDP cobinamide from adenosyl cobinamide phosphate; and the last enzyme, cobalamin synthase, combines these precursors to generate the vitamin of interest [23]. The metabolic pathway for vitamin B_12_ production in *Pseudomonas putida* KT2440 requires three major precursor compounds (highlighted in Figure 1): 5-Aminolevulinate, produced from the amino acid glycine; (R) 1-Aminopropan-2-ol, resulting from the glycine and threonine metabolism pathway; and, finally, DMB, which comes from riboflavin metabolism [11].

### 1.2. Industrial Production of Vitamin B_12_

The bacterium *P. denitrificans* contains all genes required for vitamin B_12_ synthesis, and they are mainly divided into two clusters [16]. The research on *P. denitrificans* has been mostly limited to more traditional strategies, such as random mutagenesis and optimization in fermentation processes [21]. In this work, *P. denitrificans*, as a standard in the vitamin industry, was used as a benchmark and as a starting point to quantitatively evaluate and compare the theoretical yields obtained against those of *P. putida* KT2440.

## 2. Materials and Methods

### 2.1. Organism Model

In this study, we used *Pseudomonas putida* KT2440 as a model organism. The first genome-scale metabolic model for *P. putida* KT2440 used constraint-based reconstruction analyses (COBRAs) to conduct in silico analyses [14]. In addition, the genus *Pseudomonas* is characterized by a high capacity for resistance to endogenous and exogenous stresses and the potential to produce many bioactive compounds [24].

### 2.2. Bioinformatic Model

We used a genome-scale metabolic model (GEM) for *P. putida* KT2440 obtained from the BIGG Models database and registered under BIGG ID: iJN1463; the model has 2153 metabolites, 2927 reactions, and 1462 genes [12]. The model was downloaded in SBML format for further use in the COBRA Toolbox tool 2.13.3 [25] in Matlab 2021b to perform downstream analyses. We applied constraint-based reconstruction analysis (COBRA) methods to predict cellular phenotypes and analyze the properties of metabolic engineering networks and systems [26].

We confirmed that the model of our strain contained the genes encoding for the metabolic pathway for vitamin B_12_ production. Furthermore, we verified the biochemical pathways reported for the strain in the Kyoto Encyclopedia of Genes and Genomes (KEGG) [23]. We compared the cobamide pathway reactions reported for *P. putida* with those reported for *P. denitrificans*, which is a standard in the industry, so it was considered a benchmark and reference for both bacteria.

The reactions that were not annotated in the BIGG model but were indeed in the genome were added to the model with the corresponding stoichiometric description using a COBRA Toolbox function (the “addReaction” function). The added reactions were cobyrinate a,c-diamide synthase (EC:6.3.5.9), aerobic 5,6-dimethyl benzimidazole synthase (EC: 1.13.11.79), and precorrin-3B synthase (EC:1.14.13.83). The curated model is available in the Supplementary Materials (Supplementary File S1). When required, we performed BLASTN in UNIPROT to confirm that the coding sequences for the enzyme in the KEGG pathway were present in the genome of *P. putida* KT2440. The characteristics of each aggregated reaction (substrates, products, directionality, and stoichiometry) were established with information from the KEGG database [23]. The reaction directionality of the model was verified using information from the BIGG database for each enzyme [12] and a COBRA Toolbox function (the “printRxnFormula” function); the resulting model, with the missing reactions added, was annotated in a systems biology markup language (SBML) format. In the process, COBRA Toolbox functions were used for the metabolic network curation steps [25]. The COBRA Toolbox supports SBML-like formats and models [27].

### 2.3. In Silico Culture Medium Conditions

For the FBA, bacterial biomass growth rates, and vitamin B_12_ yield, we used some of the compounds present in a culture medium previously described for *Escherichia coli* [4]. To define the glucose, succinate, glycine, and threonine consumption rates for the model, we considered the experimental values reported by Molina et al. [28]; for oxygen consumption, we used the values obtained in an in silico engineering analysis of *P. putida* [29]. We applied the COBRA Toolbox function “ChangeRxnBounds” to modify the lower limits of the consumption rates of the compounds present in the fermentation culture medium for vitamin B_12_ production. The values of the consumption rates of each compound used in the FBA are listed in Table 1.

### 2.4. Flux Balance Analysis (FBA) and Knockin

The flux balance analysis (FBA) is widely used to predict theoretical yields of genome-scale metabolic models. The FBA calculates the flux of metabolites through the metabolic network, thereby predicting the growth rate of the organism and the production rates of biotechnologically important metabolites [10]. The FBA optimizes the network for a given function, which can be the biomass of a reaction or the prediction of in silico values of growth. This optimization of metabolic flux values maximizes a selected objective function, such as target reactions [30]. Then, a matrix of stoichiometric coefficients is used for each reaction to estimate its flux according to a series of system restrictions [31].

In this study, the FBA was used to make quantitative estimates of bacterial biomass growth rates and vitamin B_12_ production yields. The yields obtained from the FBA tests were compared with the yield values of vitamin production in *P. denitrificans* [32]. For both knockin- and OptGene-based simulations, we analyzed the biosynthetic pathway of porphyrin metabolism in *P. putida* KT2440 in the KEGG database under the name “Porphyrin metabolism”. This scheme presents the reactions of the vitamin B_12_ metabolic pathway and the surrounding pathways that produce other compounds.

**Figure 2:**
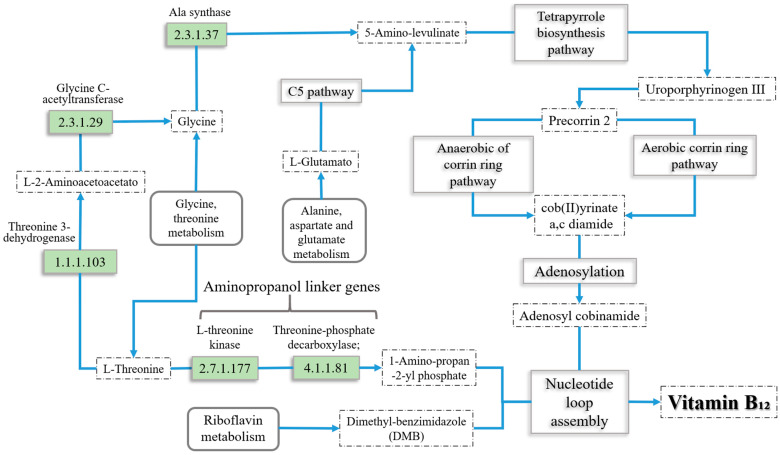
Candidate knockin-tested reactions (highlighted) added to the genome-scale metabolic model of *Pseudomonas putida* KT2440 to increase vitamin B_12_ production. Adapted from [23].

For the knockin simulations, we added to the curated model several reactions that might improve the overall production yields of vitamin B_12_ in the bacteria’s pathway. We specifically added reactions that could enhance the pathway’s precursor production, as shown in Figure 2. New reactions were added to the model with the “Add reaction” function of the COBRA Toolbox. For each knockin simulation, we estimated bacterial biomass growth and vitamin B_12_ production yields, as shown in the corresponding code (Supplementary File S2). 

### 2.5. OptGene-Based Simulations

The OptGene analysis is a technique within constraint-based in silico modeling methods such as the FBA [33,34]. It is a computational procedure consisting of a simulation of gene deletions that could increase the production rate of the objective reaction by eliminating some potentially dispensable reactions from the model. In this case, vitamin B_12_ production may be increased by reducing the number of precursors and energy invested in the synthesis of secondary or nonvital biochemical compounds and processes [34].

In the biosynthetic pathway of porphyrin metabolism in *P. putida* KT2440 in the KEGG database, we identified reactions close to the metabolic pathway of interest, specifically some that could consume part of the precursors necessary for the synthesis of vitamin B_12_. We defined a list of candidate reactions for deletion simulations, as shown in Table 2. All of the annotated reactions were included as candidates for a hypothetical knockout that could eventually increase the objective reaction, in this case, vitamin B_12_ production (EC: 2.7.8.26). We established an OptGene-based algorithm that used all possible theoretical combinations of reaction deletions, ranging from one to a maximum of five simultaneous deletions from the reaction list (Table 2), and we conducted an FBA of both the biomass and vitamin B12 functions in each simulation. The OptGene-based assay was performed by using the theoretical medium conditions described in Table 1 and the curated model with the two reactions suggested by the results of the knockin simulations (aminopropanol linker reactions). From 2 all the knockin simulations, this model with the aminopropanol linker reactions, had the best performance (see Table 3), so it was used for OptGene analyses. The corresponding code used for the OptGene-based simulations can be found in the Supplementary Materials (Supplementary File S3).

### 2.6. Identification of Riboswitches in Vitamin B_12_ Pathway

We used the Rfam database as a source of information to verify the presence of vitamin B_12_-related riboswitch sequences in the genome of *P. putida* KT2440. As the Rfam database has noncoding RNA families represented by multiple alignments, it contains useful information on secondary structures, as well as other information that can be used for computational models [18]. We searched for riboswitch sequences reported for *P. putida* KT2440 related to the vitamin B_12_ pathway.

## 3. Results

### 3.1. The Reference Value of Pseudomonas denitrificans

In a recent review, several strains were used for vitamin B_12_ production via fermentation [11], among them, the highest yield was reported for *Pseudomonas denitrificans* SC510 [32]. In some experiments on the synthesis of vitamin B_12_, several conditions of the fermentation medium of *Pseudomonas denitrificans* were modified, and, by maintaining a pH between 7.15 and 7.30, seven days (168 h) of fermentation resulted in a vitamin B_12_ concentration of 214 mg/L and a maximum dry cell mass (DWC) of 33.23 g/L. This was carried out in a medium containing sucrose, betaine, and DMB [32]. A dimensional analysis showed that this yield was equivalent to 2.83 × 10^−2^ µmol·gDW^−1^·h^−1^ (millimoles per gram of dry mass per hour). This value constitutes a benchmark for comparing the theoretical yields of the metabolic model of *P. putida* KT2440 (Figure 2).

### 3.2. Metabolic Model Curation and FBA of Pseudomonas putida KT2440

Genome-scale metabolic model curation was carried out for the porphyrin metabolism pathway. It has previously been confirmed that, at the molecular level, *P. putida* has the metabolic pathway genes for vitamin B_12_ synthesis [35]. All enzymatic reactions of the pathway of interest reported in the KEGG database were in the SBML model of *P. putida* KT2440, except for some enzymatic reactions (EC: 2.5.1.17, EC: 6.3.5.9, EC:1.13.11.79, and EC:1.14.13.83) described in the Discussion; thus, they were added to the curated model.

For the first FBA test using the curated model (the BIGG model with the missing reactions added) with the consumption rates described in Table 1 in the Section 2, we obtained a value of 1.802 gDW^−1^·h^−1^·L^−1^ for the cell biomass target function. Furthermore, by setting the last reaction (adenosylcobinamide-GDP ribazoltransferase (EC: 2.7.8.26)) of the pathway as the target function, the FBA resulted in a synthesis of 0.359 µmol gDW^−1^ h^−1^ L^−1^. This value corresponds to the theoretical production of vitamin B_12_ by *P. putida* KT2440 in the culture medium specified.

### 3.3. Gene Knockin Analysis

We performed an in silico test to evaluate the effect of the insertion of external genes, which could increase the yield of vitamin B_12_, into the *P. putida* genome. The candidate reactions tested in the metabolic model are represented in Figure 2. By considering the metabolic pathway described in Figure 1 and the list of annotated genes in Table 3, the insertion of ALA synthase (EC: 2.3.1.37) from the C4 pathway for 5-amino-levulinate synthesis was assessed. We also considered two reactions from glycine, serine, and threonine metabolism as candidates for insertion because of their potential to increase the production of the precursor aminopropan-2-ol. Specifically, we considered the threonine 3-dehydrogenase (EC: 1.1.1.103) and the glycine C-acetyltransferase (EC: 2.3.1.29) for this purpose. The two reactions responsible for the aminopropanol linking part, threonine kinase (EC: 2.7.1.177) and threonine phosphate decarboxylase (EC: 4.1.1.81), were also established as candidates. The results of the FBA performed with different reaction candidates are shown in Table 3.

The biomass yields stayed the same for all the models (1.802 gDW^−1^·h^−1^·L^−1^), which suggests that the insertion of any of these candidate reactions is not detrimental to bacterial growth.

### 3.4. OptGene Analysis

The OptGene-based algorithm was used to evaluate all the combinations of reaction deletions possible for our 12 reaction candidates (Table 2), ranging from one to a maximum of five different reaction deletions, resulting in a total of 1585 simulations. We conducted 12 simulations for single reaction deletions (one for each reaction), 66 simulations for sets of two reaction deletions, 220 simulations for sets of three reaction deletions, 495 simulations for sets of four reaction deletions, and 792 simulations for sets of five reaction deletions. The vast majority of the simulations resulted in biomass growth or vitamin B_12_ production values of zero or very close to zero. The highest values for both biomass growth and vitamin B_12_ were, at best, the same or very similar to those obtained when using the curated model with the aminopropanol linker reactions added. The highest value obtained for vitamin B_12_ yield was only 0.3% higher than that obtained with our model with the aminopropanol linker reactions, with the biomass growth performance being the same; this simulation case required the deletion of four different reactions (EC: 1.3.98.3; EC: 1.4.4.2; EC: 1.8.1.4/1.2.4.2; and EC: 1.1.1.381). The specific values of each simulation can be found in the Supplementary Materials (Supplementary File S4). Therefore, our algorithm suggests that our candidate reactions do not necessarily work as competitors for precursors or energy in the biosynthetic pathway of vitamin B_12_ synthesis. This evidence suggests that none of the candidate gene deletions should be carried out to optimize vitamin B_12_ production in *P. putida* KT2440.

### 3.5. Theoretical Performance of Pseudomonas putida KT2440 versus Experimental Results of Pseudomonas denitrificans

**Figure 3:**
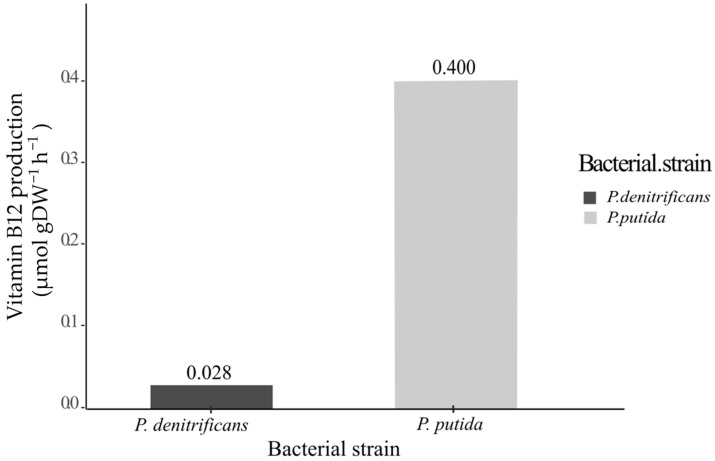
Experimental production of vitamin B_12_ in *Pseudomonas denitrificans* SC510 [32] and theoretical production of vitamin B_12_ in modified Pseudomonas putida KT2440 according to the metabolic FBA of a BIGG model (BIGG ID: iJN1463), subsequently curated with the insertion of the aminopropanol linker reaction and under the culture and substrate consumption conditions described in Table 1.

We used *P. denitrificans* SC510 as a point of comparison to validate the feasibility of *P. putida* KT2440 as a vector for vitamin B_12_ production. For this purpose, we used the results of the metabolic model with the aminopropanol linker genes under the medium conditions described in Table 1 in the Section 2 in support of previous studies [4,29,36]. Figure 3 shows that the theoretical vitamin B_12_ production yield obtained for the modified *P. putida* KT2440 (0.400 µmol gDW^−1^ h^−1^ L^−1^) is higher than the experimental yield obtained for *P. denitrificans* SC510 [32].

### 3.6. Riboswitches Identified in the Vitamin B_12_ Pathway

In *Pseudomonas putida* KT2440, we detected five putative vitamin B_12_ riboswitches according to Rfam, as described in Table 4, all with the same Rfam accession (RF00174). All the sequences were also confirmed in the NCBI as being part of *P. putida* KT2440.

## 4. Discussion

### 4.1. Biosynthetic Pathway of Vitamin B_12_ in Pseudomonas denitrificans and Pseudomonas putida

Oxygen-dependent vitamin B_12_ synthesis is the predominant biochemical pathway in Proteobacteria that synthesizes the molecule. In the case of *P. putida*, the presence of genes encoding for the vitamin B_12_ synthesis pathway has been verified. The set of reactions reported in *P. putida* is similar to that reported in *P. denitrificans*, as shown in Table 5 [35]. *P. putida*, as a strictly aerobic organism [29], is also expected to use the aerobic route of corrin ring synthesis (Figure 1). The similarity of the vitamin B_12_ synthesis reactions in the two *Pseudomonas* species assessed is shown in Table 5. Of the 38 reactions named, *P. denitrificans* has 31, and *P. putida* has 32; in the vitamin B_12_ pathway, they differ only in the enzyme precorrin-6A synthase, also called CobF (EC:2.1.1.1.152), which is absent in *P. denitrificans* [35]. However, both strains lack the two aminopropanol binding reactions, which are responsible for transforming L-threonine into 1-aminopropan-2-ol. Phosphate is incorporated in one of the last steps of vitamin B_12_ synthesis [23].

### 4.2. Genome-Scale Metabolic Models of Pseudomonas putida KT2440

Genome-scale metabolic models (GEMs) describe gene–protein reaction processes for all the sequences that participate in metabolic reactions. GEMs can help to predict metabolic fluxes for a given pathway using optimization techniques such as the flux balance analysis (FBA) [37]. GEMs of different organisms have been used in industrial, medical, and scientific applications. These models have been used for the prediction of effective gene engineering strategies to enhance the microbial production of certain compounds and materials [37].

According to the biosynthetic pathway gene annotation shown in Table 5 it is important to highlight that although the biochemical vitamin B_12_ pathway in *Pseudomonas* has been described to be oxygen-dependent, genes for the reactions of the anaerobic pathway are reported in both strains [35]. When verifying the reactions in Table 2 in the BIGG Database metabolic model of *P putida* KT2440, we observed that genes reported as being part of the anaerobic pathway were not annotated as such in the iJN1463 model obtained from BIGG. Regarding the anaerobic reactions reported in Table 5, their aerobic equivalents were in the model, as the KEGG database showed both EC codes (anaerobic and aerobic) for the same reaction [23]. It is possible that the anaerobic pathway’s annotation was assigned by homology or by the annotation algorithm. This hypothesis makes sense, as a strictly aerobic bacterium such as *P. putida* [29] should not require genes or reactions in the anaerobic pathway of corrin ring synthesis.

Another important aspect is that, in Table 5, the last reaction in the aerobic pathway of corrin ring synthesis (EC: 1.16.8.1), which is not reported for *Pseudomonas*, actually corresponds to the enzyme EC: 2.5.1.17, which belongs to the next step, adenosylation. This reaction was confirmed in the KEGG database, where the EC code for the reaction was updated [23]. Furthermore, the EC: 6.3.5.9 and EC:1.13.11.79 reactions were indeed in the genome of *P. putida* KT2440 (confirmed by BLAST) but were not annotated in the BIGG model, so we added them with the “Add reaction” function of the COBRA Toolbox. We noted that the precorrin-3B synthase reaction (EC:1.14.13.83) was in the model but not properly named, so it was also added by using the same function. The aminopropanol dehydrogenase reaction (EC 1.1.1.75) was not reported in the KEGG map but was in the BIGG model. The anaerobic reaction EC:2.1.1.195 is present in the genome of *P. putida* KT2440 but was not annotated in the model obtained.

### 4.3. Gene Knockin Analysis

The biomass yields were the same for all the models (1.802 gDW^−1^·h^−1^·L^−1^), which implies that the insertion of any of these candidate reactions is not detrimental to bacterial growth. These results suggest that the introduced reactions mainly affect the metabolic pathway of vitamin synthesis and not necessarily the core metabolism of *P. putida* KT2440. Individually, both aminopropanol linker reactions and Ala synthase (alone or with Glycine C-acetyltransferase) resulted in higher vitamin B_12_ production yields, with 0.400 and 0.394 µmol gDW^−1^·h^−1^·L^−1^ of vitamin B_12_, respectively. None of the other models surpassed the vitamin B_12_ production of the first model (the curated model), even presenting a significant decrease in the predicted production rate. After adding both the ALA synthase reaction and the aminopropanol linker reactions, the FBA values for the biomass function stayed the same, but vitamin B_12_ synthesis decreased significantly. Interestingly, the combination of these reactions in the same model led to a decrease in vitamin B_12_ production.

The ALA synthase reaction (EC: 2.3.1.37) is a central part of the first stage of synthesis, specifically in the C4 pathway, in which it produces 5-amino-levulinate from glycine and succinyl-CoA. Conversely, the aminopropanol linker reactions use L-threonine for the production of (R) 1-Amino-propan-2-yl phosphate. Both ALA synthase and the amipropanol linker pathways obtain their substrates from glycine and threonine metabolism [11]. This may explain why having both pathways working at the same time reduces the overall production of vitamin B_12_, as they may compete with each other for precursor compounds. A genetic modification strategy that involves the insertion of any of these two options should include one but not both of them at the same time.

According to this result, we suggest introducing aminopropanol linker genes into the organism as part of a genetic construct to optimize vitamin B_12_ production in *P. putida* KT2440. The insertion of these genes could increase vitamin B_12_ production by approximately 11.4% compared with the bacterial model without genetic modifications. If so, it may be necessary to complement this genetic strategy with other methods, such as gene overexpression and an adequate culture medium.

According to our results, the theoretical vitamin B_12_ synthesis rate calculated using *P. putida* KT2040 with the addition of aminopropanol linker genes was about 14 times higher than that of *P. denitrificans*, and the cell biomass growth rate reached a value of 1.802 gDW^−1^·h^−1^·L^−1^. Although more factors can be considered to make more accurate predictions, this in silico analysis result indicates that *P. putida* KT2440 is favorable as a production system.

As stated in previous studies on *P. denitrificans* for vitamin B_12_ production, it is important to consider the presence of riboswitches in the bacterial genome [38]. B_12_ riboswitches may be present in the 5′ nontranscribed regions of mRNA and may form a secondary structure that senses vitamin B_12_ upon binding to it. It is one of the control mechanisms most predominant in the metabolism of vitamin B_12_ [39]. B_12_ riboswitches are RNA-sensitive control elements in the cis-regulatory region and modulate gene expression in many vitamin B_12_-producing microorganisms. They function as ligand-responsive control elements [38]. Considering the five putative B_12_ riboswitches detected according to Rfam, we propose that modifying this regulatory mechanism can increase the yield of vitamin B_12_ synthesis in *P. putida* KT2440. This modification may be achieved by using genetic editing tools such as CRISPR-Cas9. When vitamin B_12_ increases, its high concentrations may favor the sequestration of ribosome binding sites and, thus, block the translation initiation of pathway enzymes [40]. Although B_12_ riboswitches can present considerable challenges, it was recently demonstrated that all riboswitches in Cbl Cluster I in *Pseudomonas* ATCC 13867 could be completely removed. Furthermore, the promoters regulated by those riboswitches were replaced by strong constitutive promoters that doubled B_12_ biosynthesis in the strain [16]. Therefore, we consider that these riboswitches detected in the KT2440 strain are possible targets for genetic engineering, such as CRISPR/Cas9-mediated genome editing, to improve the expression of genes involved in vitamin B_12_ synthesis.

### 4.4. Design of Possible Genetic Construct for Vitamin B_12_ Optimization in Pseudomonas putida KT2440

**Figure 4:**
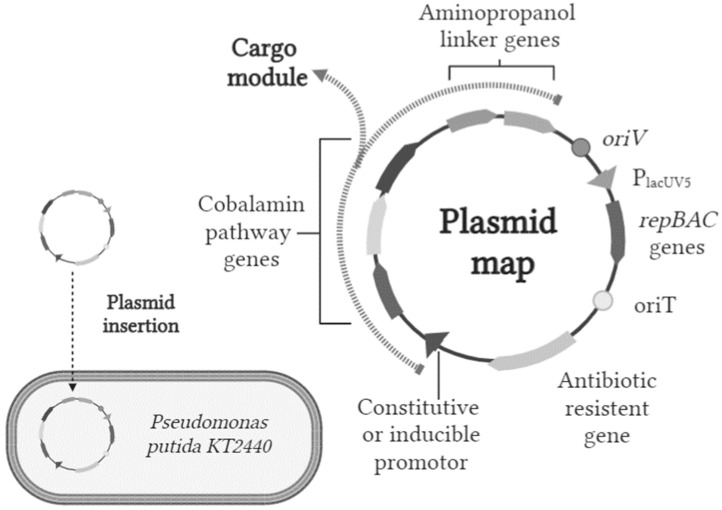
Possible genetic modification strategy for the optimization of *Pseudomonas putida* KT2440 vitamin B_12_. One possible strategy for vitamin B_12_ optimization may consist of including both aminopropanol linker genes and changing the riboswitches of the vitamin B_12_ pathway to constitutive promoter sequences. Data from [40].

According to the results obtained in silico, we propose a genetic modification strategy for increasing vitamin B_12_ production in *P. putida* KT2440. The genetic modification strategy consists of the addition of aminopropanol linker genes and the modification of riboswitches related to vitamin B_12_ enzyme sequences. Figure 4 shows the general structure of the plasmid that could be inserted into *P. putida* KT2440; the general design and some sequences belong to the Standard European Vector Architecture (SEVA) Database [41]. Besides the “Cargo module” (where sequences of interest are inserted), which contains the promoters and genes of the vitamin B_12_ pathway and the genes of the aminopropanol linker that code for enzymes, as expected, the plasmid should include housekeeping components such as the origins of replication and antibiotic resistance. We believe that changing the riboswitches of the vitamin B_12_ pathway genes is crucial for optimizing our bacterial strain for vitamin B_12_ production, as suggested by studies on *P. denitrificans* [16].

The housekeeping components of the plasmid contain the *oriV*, *P_lacUV5_*, and *repBAC* genes and *oriT* components. The *oriV* segment is a minimum sequence for replication initiation and may encode replication proteins. The *repBAC* genes constitute replication proteins that often follow *oriV* and are preceded by the *P_lacUV5_* promoter. These replication origin sequences come from the RSF1010 replicon of the IncQ plasmid. The host range of IncQ plasmids includes pseudomonads and other proteobacteria relatives, and it has been widely used to create a large number of plasmid vectors. The *oriT* element allows for the conjugative mobilization of the plasmid to the organism, especially in organisms lacking alternative transformation methods [41]. In the antibiotic resistance gene, the sequence may vary between the most standardized markers for selection in Gram-negative bacteria, and there are several options, such as ampicillin, kanamycin, chloramphenicol, streptomycin, tetracycline, and gentamicin [41]. We expect this plasmid insertion strategy to increase the yields of vitamin production in *P. putida* KT2440. The specific identity of some components can be defined and modified in the process according to technical criteria.

## 5. Conclusions

The strain *Pseudomonas putida* KT2440 has significant potential for the synthesis of valuable organic compounds in general terms and specifically for vitamin B_12_. It has all the qualities necessary to serve as an appropriate production system for biotechnological purposes, and, as it has reactions for the synthesis of vitamin B_12_, it is an optimal candidate for the industrial production of this compound. The theoretical yields obtained using flow balance analysis (FBA) tests suggest that this proteobacterium, with the addition of aminopropanol linker genes and an optimized culture medium, could be a great producer of vitamin B_12_. Furthermore, the results of OptGene-based simulations suggest that all native reactions should be maintained in the model. Due to the presence of riboswitches in the vitamin B_12_ pathway, some genetic modifications of regulatory sequences may improve gene expression and, subsequently, vitamin B_12_ production. However, incorporating constitutive promoters into vitamin B_12_ gene clusters will be more convenient than modifying riboswitch sequences.

It is important to highlight that the in silico analyses performed did not consider molecular regulation, such as the negative feedback that can occur in the transcription or translation of the sequences of the biochemical pathway. In this regard, it is necessary to thoroughly verify the presence of regulatory sequences, such as riboswitches, that could limit vitamin B_12_ synthesis. Further bioinformatics analyses and laboratory experiments could provide even more information on other genetic engineering strategies that could be implemented.

## Figures and Tables

**Table 1:** Consumption rates of compounds from the fermentation medium for vitamin B_12_ synthesis.

Compound	Consumption Rates (mmol/gdw·h)
Oxygen	18.5
Glucose	11.7
L-Threonine	0.48
Succinate	0.95
Glycine	0.1
Cobalt^2+^	0.0375

**Table 2:** List of reactions used for the OptGene test.

	Reaction Name	EC Number
1	Coproporphyrinogen oxidase	1.3.3.3
2	Protoporphyrinogen oxidase (aerobic)	1.3.3.4
3	Oxygen-independent coproporphyrinogen III dehydrogenase	1.3.98.3–1.3.99.22
4	Uroporphyrinogen decarboxylase (uroporphyrinogen III)	4.1.1.37
5	Sirohydrochlorin ferrochelatase	4.99.1.4
6	Ferrochelatase	4.99.1.1
7	Glycine cleavage system	1.4.4.2
8	Tetrahydrofolate aminomethyltransferase	2.1.2.10
9	Aminomethyltransferase	2.1.2.10
10	2-Oxogluterate dehydrogenase	1.8.1.4–1.2.4.2
11	L-allo-threonine dehydrogenase	1.1.1.381
12	Sirohydrochlorin dehydrogenase (NAD)	1.3.1.76

**Table 3:** Results of flux balance analysis rates of biomass and vitamin B_12_ production for the *Pseudomonas putida* KT2440 genome-scale metabolic models with the candidate knockin reaction(s) added. All values were obtained with the culture and substrate consumption conditions described in Table 1.

Name of the Reaction(s) Added to the Model	EC Numbers of Reaction(s) Added	Vitamin B_12_ Production (µmol gDW^−1^ h^−1^ L^−1^)
None (curated model)	-	0.359
Aminopropanol linker	2.7.1.177, 4.1.1.81	0.400
Ala synthase reaction	2.3.1.37	0.394
Ala synthase reaction and Glycine C-acetyltransferase	2.3.1.37 and 2.3.1.29	0.394
Aminopropanol linker and Ala synthase	2.7.1.177, 4.1.1.81 and 2.3.1.37	0.215
Threonine 3-dehydrogenase	1.1.1.103	0.230
Glycine C-acetyltransferase	2.3.1.29	0.180
Threonine 3-dehydrogenase and glycine C-acetyltransferase	1.1.1.103, 2.3.1.29	0.391

**Table 4:** Information of vitamin B_12_ regulatory riboswitches in *Pseud omonasputida* KT2440.

Riboswitch	Length	Position	Rfam Accession
1	207	2,768,769–2,768,976	RF00174
2	222	3,857,546–3,857,768	RF00174
3	197	2,765,029–2,765,226	RF00174
4	205	398,802–3,982,007	RF00174
5	220	1,866,938–1,867,158	RF00174

**Table 5:** Cobamide biosynthetic pathway gene annotation in *Pseudomonas denitrificans* and *Pseudomonas putida.* Adapted from [35].

	*Pseudomonas* *denitrificans*	*Pseudomonas putida*	EC Number of Reported Reactions	Stage of Biosynthesis
1	X	X	Síntesis de ALA (HemA o HemAL)	
2	X	X	EC:4.2.1.24 (HemB)	Tetrapyrrole
3	X	X	EC:2.5.1.61 (HemC)	Precursor
4	X	X	EC:4.2.1.75 (HemD)	Biosynthesis
5	X	X	EC:2.1.1.107/4.99.1.4 (CysG/CobA)	
6	X	X	EC:1.3.1.76/4.99.1.4/2.1.1.107 (CysG)	
7	-	-	EC:4.99.1.3 (CbiK/CbiX)	
8	X	X	EC:2.1.1.151 (CbiL)	
9	X	X	EC:2.1.1.131 (CbiH/CobJ)	
10	X	X	EC:2.1.1.271/2.1.1.133 (CbiF/CobM)	
11	X	X	EC:3.7.1.12/2.1.1.131 (CbiG/CobJ)	
12	X	X	EC:2.1.1.195 (CbiD)	Anaerobic
13	X	X	EC:1.3.1.106/1.3.1.54 (CbiJ/CobK)	Corrin Ring
14	-	-	EC:2.1.1.196/2.1.1.289/2.1.1.132(CbiT/CobL)	Biosynthesis
15	-	-	EC:2.1.1.289/2.1.1.132 (CbiE/CobL)	
16	X	X	EC:5.4.99.60/5.4.99.61 (CbiC/CobH)	
17	X	X	EC:6.3.5.11/6.3.5.9 (CbiA/CobB)	
18	X	X	EC:2.1.1.130 (CobI)	
19	X	X	EC:1.14.13.83 (CobG)	
20	X	X	EC:2.1.1.131 (CobJ)	
21	X	X	EC:2.1.1.133 (CobM)	
22	-	X	EC:2.1.1.152 (CobF)	Aerobic
23	X	X	EC:1.3.1.54 (CobK)	Corrin Ring
24	X	X	EC:2.1.1.132 (CobL)	Biosynthesis
25	X	X	EC:5.4.99.61 (CobH)	
26	X	X	EC:6.3.5.9 (CobB)	
27	X	X	EC:6.6.1.2 (CobNST)	
28	-	-	EC:1.16.8.1/2.5.1.17 (CobR/pduO)	
29	X	X	EC:2.5.1.17 (CobA/BtuR/CobO/PduO)	Adenosylation
30	-	-	EC:2.7.1.177 (PduX)	Aminopropanol
31	-	-	EC:4.1.1.81 (CobD)	Linker
32	X	X	EC:6.3.5.10 (CbiP/CobQ)	
33	X	X	EC:6.3.1.10 (CbiB/CobC/CobD)	
34	X	X	EC:2.7.1.156/2.7.7.62 (CobU/CobP)	Nucleotide Loop
35	X	X	EC: 2.7.7.62 (CobU/CobP/CobY)	Assembly
36	X	X	EC:2.4.2.21 (CobT/CobU/ArsAB)	
37	X	X	EC:3.1.3.73 Cbl Fosfatasa (CobS/CobV)	
38	X	X	EC:2.7.8.26 (CobC/CobZ)	

X represents the presence of the gene.

## Data Availability

The original contributions presented in the study are included in the article and Supplementary Materials, further inquiries can be directed to the corresponding author/s.

## Supplementary Materials

The following supporting information can be downloaded at https://www.mdpi.com/article/10.3390/metabo14110636/s1, Supplementary File S1: Curated model of Pseudomonas putida KT2440, Supplementary File S2: Flux Balance Analysis and Knockin simulations, Supplementary File S3: OptGene-based simulations, Supplementary File S4: Optgene-based simulations’ results.
